# Long-term survival without surgery following a complete response to pre-operative chemoradiotherapy for rectal cancer: A case series

**DOI:** 10.3892/ol.2013.1596

**Published:** 2013-10-01

**Authors:** SEUNG-GU YEO, DAE YONG KIM, JAE HWAN OH

**Affiliations:** 1 Department of Radiation Oncology, Soonchunhyang University College of Medicine, Cheonan, Chungnam 330-721, Republic of Korea; 2 Center for Colorectal Cancer, Research Institute and Hospital, National Cancer Center, Goyang, Gyeonggi 410-769, Republic of Korea

**Keywords:** rectal cancer, pre-operative chemoradiotherapy, clinical complete response, wait-and-see

## Abstract

Pre-operative chemoradiotherapy (CRT) for rectal cancer yields a complete tumor response in 10–30% of patients. There is an argument for omitting surgery in these patients, but this remains highly controversial and the supporting evidence based on long-term follow-up is lacking. The present study analyzed the long-term outcomes of five patients with cT3 or cT4 rectal cancer who showed a clinical complete response (ycCR) following pre-operative CRT and underwent no surgery. The ycCR status was determined 7–12 weeks after the completion of CRT using clinical, endoscopic and radiological studies, including magnetic resonance imaging and biopsy. The follow-up period was 54–101 months. Three patients had no tumor recurrence and were alive with no evidence of disease at 101, 100 and 93 months, respectively. One patient developed local recurrence at 59 months and another developed lung metastasis at 32 months. The two patients with tumor recurrence remained disease-free 42 and 22 months after salvage pelvic and thoracic surgery, respectively. Despite being a small series, the long-term survival outcomes of the present study indicate that a non-operative approach may be feasible for a proportion of rectal cancer patients who reveal a ycCR following pre-operative CRT.

## Introduction

Chemoradiotherapy (CRT) prior to surgery has become the preferred treatment approach for patients with locally-advanced rectal cancer (LARC). In addition to improving local disease control, pre-operative CRT leads to significant tumor regression (downsizing) and a shift toward a lower stage (downstaging), in the primary tumor and perirectal lymph nodes. In 10–30% of patients, the specimens resected during radical surgery reveal no residual cancer cells, i.e. a post-CRT pathological complete response (ypCR) (1). For these who have markedly radiosensitive tumors, the cancer cells are not present at the time of surgery and thus patients may be overtreated and exposed unnecessarily to the risks of major pelvic surgery, including disorders of urinary, fecal and sexual functions, stoma formation and even surgical mortality.

Therefore, patients with significant or complete tumor regression following pre-operative CRT may undergo alternative treatment strategies, including transanal local excision, or even no immediate surgery with strict follow-up (2). Habr-Gama *et al* proposed the ‘wait-and-see’ strategy based on the observation of no survival benefit in patients with a confirmed ypCR through radical surgery over patients with a clinical complete response (ycCR) who did not undergo any surgical management (3). Maas *et al* reproduced the favorable results of this wait-and-see policy following a post-CRT ycCR (4). Nevertheless, this approach remains highly controversial and supporting evidence based on long-term follow-up is lacking (5).

The present study reports the long-term outcomes of five patients with LARC who were managed with pre-operative CRT only, without any surgical resection, following a ycCR to pre-operative CRT.

## Materials and methods

### Patients

A total of 577 patients with LARC received pre-operative CRT between 2004 and 2008 at the National Cancer Center (Goyang, Korea). Among them, five patients who had a ycCR following pre-operative CRT and underwent no surgery were analyzed retrospectively. All patients had biopsy-proven adenocarcinoma of the middle or lower rectum and clinical T3 or T4 tumors on magnetic resonance imaging (MRI). The wait-and-see approach was presented to the patients as experimental and it was stressed that radical surgery following pre-operative CRT is the standard oncological treatment. Five patients selected this policy, largely due to the possibility of avoiding major surgery or a permanent stoma. The pre-treatment staging work-up included a digital rectal examination, complete blood count, liver function tests, serum carcinoembryonic antigen level measurements, video colonoscopy, chest radiography, computed tomography (CT) of the abdomen and pelvis and MRI with or without transrectal ultrasonography. Clinically-positive lymph node involvement was defined as a lymph node of ≥0.5 cm in the short-axis diameter observed on MRI or CT. This study was performed in accordance with the guidelines of the Institutional Review Board of the National Cancer Center (Goyang, Korea) and informed consent was obtained for each patient.

### Treatments

A dose of 45 Gy pre-operative radiotherapy was delivered to the whole pelvis in 25 fractions, followed by a 5.4 Gy boost in three fractions within 6 weeks. Each patient underwent CT simulation for three-dimensional conformal radiotherapy planning and a three-field treatment plan that consisted of a 6-MV photon posterior-anterior field and 15-MV photon opposed lateral beams. The gross tumor volume, mesorectum, presacral space, entire sacral hollow and regional lymphatics, including perirectal, internal iliac, presacral and distal common iliac lymphatics were encompassed by the initial radiation field. The boost field included the gross tumor volume and mesorectum with ≥2-cm margins in all directions.

The pre-operative chemotherapy administered concurrently with radiotherapy was one of the following three regimens (Table I): i) Two cycles of intravenous bolus injections of 400 mg/m^2^/day 5-fluorouracil and 20 mg/m^2^/day leucovorin for 3 days in the first and fifth weeks of radiotherapy; ii) 825 mg/m^2^ oral capecitabine twice daily during radiotherapy without weekend breaks; or iii) 825 mg/m^2^ oral capecitabine twice daily during radiotherapy with weekend breaks and 40 mg/m^2^/day intravenous irinotecan during each week of radiotherapy. Post-CRT adjuvant chemotherapy was delivered in one patient; four 5-week cycles with each cycle consisting of 400 mg/m^2^/day UFT-E (tegafur-uracil) plus 90 mg/day leucovorin for 4 weeks followed by a 1-week rest.

### Evaluation and follow-up

**Figure 1:**
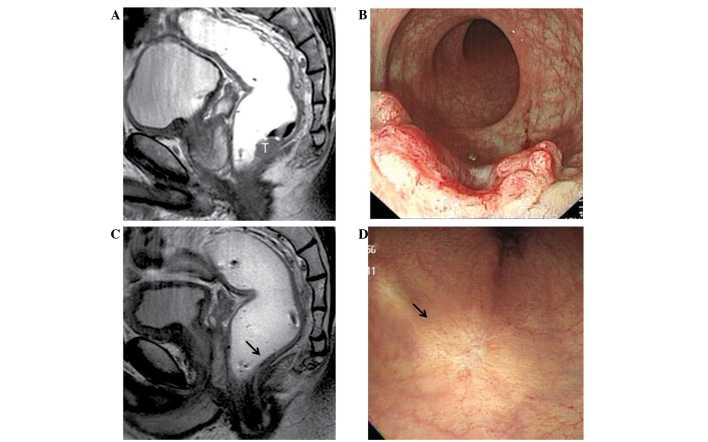
An example of a post-chemoradiotherapy ycCR. (A and B) Pre-treatment tumor (T) in sagittal T2-weighted MRI and endoscopy, respectively. (C and D) Post-chemoradiotherapy sagittal T2-weighted MRI and endoscopy, respectively. (C) Arrow indicates hypointense bowel wall, indicative of fibrosis; (D) arrow indicates whitening of the mucosa. ycCR, clinical complete response; MRI, magnetic resonance imaging.

The tumor response was assessed 7–12 weeks after the completion of CRT using the same clinical, endoscopic and radiological studies as for the initial work-up. The decision criteria regarding a ycCR included: i) No palpable tumor or stenosis on digital rectal examination; ii) no residual intraluminal mass or ulceration at endoscopy; iii) no residual mural tumor and suspicious lymph nodes on MRI or CT; and iv) a negative biopsy. The primary rectal tumor site was biopsied following CRT in four patients. In one patient, the biopsy was conducted at 19 weeks following CRT, as the rectal tumor, located within the peritoneal cavity, showed massive necrosis and there was concern regarding bowel perforation upon biopsy. Fig. 1 shows an example of a pre-treatment tumor and the post-CRT status of ycCR.

The patients were followed up every 3 months for the first 2 years, every 6 months for the next 3 years and annually thereafter. The follow-up evaluations consisted of a physical examination, complete blood count, liver function tests and serum carcinoembryonic antigen level measurement at each visit. Chest radiography and CT scanning of the abdomen and pelvis were conducted every 6 months for 5 years and annually thereafter. Video colonoscopy or sigmoidoscopy was performed every year.

## Results

### Patient demographics

The patient demographics, treatments and outcomes are presented in Table I. Patient age ranged between 52 and 69 years old. All of the patients were male. The distal end of the tumor was located 1–9 cm from the anal verge. The clinical T classification was T3 in four patients and T4 in one (ureter invasion) patient.

### Follow-up

The follow-up period ranged from 54–101 months. Three patients had no tumor recurrence as of the last follow-up and were alive with no evidence of disease at 101, 100 and 93 months, respectively. Patient 2 developed local recurrence where the primary tumor was located initially at 59 months. A low anterior resection was performed, revealing a moderately-differentiated adenocarcinoma that had invaded through the muscularis propria into the perirectal tissue. Six retrieved lymph nodes were all negative and the circumferential resection margin was clear (0.4 cm). The patient refused the recommended post-operative chemotherapy. Patient 5 developed lung metastasis at 32 months. This individual received induction chemotherapy consisting of nine cycles of 5-fluorouracil, leucovorin and oxaliplatin (FOLFOX), then wedge resection surgery and six post-operative cycles of FOLFOX. The surgical specimen contained a 0.7-cm metastatic adenocarcinoma. These two patients with disease recurrence remained disease-free 42 and 22 months after salvage pelvic and thoracic surgery, respectively.

## Discussion

The wait-and-see strategy of close observation without surgery was proposed by Habr-Gama *et al* for selected rectal cancer patients who achieve a ycCR following pre-operative CRT (6). The authors compared long-term outcomes between 71 patients who were managed with this strategy and 22 patients who had a post-surgery ypCR. The clinical assessment was conducted 8 weeks after the completion of CRT, and only patients who sustained this ycCR status until 1 year were selected. The 5-year overall and disease-free survival rates were 88 and 83%, respectively, in the resection group, and 100 and 92%, respectively, in the observation group (3). The updated study by this group analyzed 99 patients with a sustained ycCR, and the 5-year overall and disease-free survival rates were 93 and 85%, respectively (7). These favorable outcomes were similar to those recorded by Yeo *et al*, who reported 5-year overall and disease-free survival rates of 94.8 and 88.5%, respectively, in 304 LARC patients with post-CRT ypT0N0 with radical surgery (1). Recently, an additional institution also reported no difference in outcomes between 21 ycCR LARC patients treated with this approach and patients with post-surgery ypCR. However, the follow-up period was only 2 years (4). In a meta-analysis of rectal cancer, late recurrence presenting >5 years after the initial therapy constituted 24% of all local recurrences when pre-operative long-course CRT or radiotherapy was performed (8). The favorable outcomes following long-term follow-up in the present study, although in a small series, may constitute additional supporting evidence for this wait-and-see strategy. All five patients were alive with no disease at the last follow-up, which was ~8 years since CRT completion in four patients.

However, this approach remains highly controversial and several limitations, which appear to be difficult to overcome, have held back its widespread adoption. The major obstacle preventing this policy from gaining popularity is the difficulty distinguishing between residual cancer and CRT-induced fibrosis, clinically or radiologically without using surgical pathology. Habr-Gama *et al* defined a ycCR as the absence of a residual mass or ulcer on digital examination and endoscopy, no signs of residual tumor observed in radiological studies (CT and ultrasound) and a negative biopsy. Since these methods are inevitably limited in terms of objective and accurate identification of a ycCR, the patients were followed up monthly for 1 year, at which time, clinical complete responders were determined (3,7). In addition to using modalities and criteria similar to those above, MRI was used in the initial work-up and for assessing the tumor response. Patients who maintained a ycCR for 1 year were not the only individuals selected in the present study; however, recurrences did not occur <2 years after CRT. Maas *et al*, who first reproduced the results of this strategy, used more sophisticated modalities, including MRI enhanced with novel contrast agents or diffusion-weighted MRI (4). Restaging MRI, consisting of standard T2-weighted MRI and diffusion-weighted MRI, significantly improved the sensitivity for selecting complete responders, with a specificity of >90%; i.e. the risk of underestimating the residual tumor was <10% (9). In the more recent ACOSOG Z6041 trial, which investigated the efficacy of pre-operative CRT and local excision for treating cT2N0 rectal cancer, a ycCR was concordant with a ypCR in 31 of 36 patients (10). There has been an attempt to standardize the clinical and endoscopic observations for defining a post-CRT ycCR in rectal cancer (11), and the future development of molecular and radiological tools may improve the accurate clinical identification of such cases.

Two patients in the present series, who had disease recurrence at 59 and 32 months, were salvaged successfully and were alive with no disease 42 and 22 months following surgical resection of a recurrent local and distant tumor, respectively. Earlier local recurrence, including within an arbitrary 1 year in the study by Habr-Gama *et al*, may be attributable to a misdiagnosis of a ycCR and regrowth of the tumor. If the oncological outcome is compromised by delaying surgery in these patients, the wait-and-see strategy may have to be abandoned considering the current restricted capability for accurate identification of a ycCR. In this regard, Habr-Gama *et al* reported that patients who eventually required surgery following a suspected, but not sustained, ycCR for 1 year, did not have inferior oncological outcomes compared with those who were considered to have had an incomplete response and had undergone immediate surgery (12). The response to pre-operative CRT is a significant predictor of the oncological outcome (1,13). It has been hypothesized that prolonged intervals until surgery may have been counterbalanced by favorable biological tumor behavior leading to insignificant differences in oncological outcome compared with patients with a less radiosensitive tumor, but managed with immediate surgery (12). A comparison of the outcomes between these suspected, but not sustained, ycCR patients with ycCR patients undergoing immediate surgery is required in order to reveal whether delayed surgery results in an oncological compromise.

In an era of pursuing individualized, tailored treatment strategies, the fact that a proportion of patients with LARC develop a complete response is an advantage of a pre-operative CRT approach. Non-operative management of LARC patients with a ycCR following CRT may be feasible with strict selection criteria and frequent follow-up. The results of ongoing prospective trials of this wait-and-see policy are currently awaited (14). While sufficient evidence is accumulated, this strategy may be of specific value for the elderly and for patients with comorbidity, particularly if the planned radical surgery involves a permanent colostomy.

## Figures and Tables

**Table I:** Patient characteristics, treatments and outcomes.

Pt	Age, years	Gender	Pre-CRT CEA, ng/ml	Diff	Location from AV, cm	cStage	Conc CT	Post-CRT CEA, ng/ml	Post-CRT Bx (weeks)	Adj CT	Recurrence (months)	Current status (months)
1	63	M	2.5	M	1.0	T3N0M0	X	2.6	-	-	-	NED (101)
2	69	M	2.0	W	8.0	T3N1M0	IX	2.6	Y (12)	-	LR (59)	NED (101)
3	64	M	1.8	M	5.5	T3N1M0	IX	1.7	Y (7)	-	-	NED (100)
4	53	M	5.2	M	4.5	T3N1M0	IX	4.2	Y (8)	-	-	NED (93)
5	52	M	2.2	M	9.0	T4N2M0	FL	1.8	Y (19)	UFT	DM (32)	NED (54)

Pt, patient; CRT, chemoradiotherapy; CEA, carcinoembryonic antigen; Diff, differentiation (M, moderate; W, well); AV, anal verge; Conc CT, concurrent chemotherapy; Bx, biopsy; Adj CT, adjuvant chemotherapy; X, capecitabine; IX, irinotecan and capecitabine; FL, 5-fluorouracil and leucovorin; LR, local recurrence; DM, distant metastasis; NED, alive with no evidence of disease; Y, yes.
